# Mesenchymal Stem Cells as New Therapeutic Agents for the Treatment of Primary Biliary Cholangitis

**DOI:** 10.1155/2017/7492836

**Published:** 2017-12-19

**Authors:** Aleksandar Arsenijevic, C. Randall Harrell, Crissy Fellabaum, Vladislav Volarevic

**Affiliations:** ^1^Faculty of Medical Sciences, Department of Microbiology and Immunology, Center for Molecular Medicine and Stem Cell Research, University of Kragujevac, Kragujevac, Serbia; ^2^Regenerative Processing Plant, LLC, Palm Harbor, FL, USA

## Abstract

Primary biliary cholangitis (PBC) is a chronic autoimmune cholestatic liver disease characterized by the progressive destruction of small- and medium-sized intrahepatic bile ducts with resultant cholestasis and progressive fibrosis. Ursodeoxycholic acid and obethicholic acid are the only agents approved by the US Food and Drug Administration (FDA) for the treatment of PBC. However, for patients with advanced, end-stage PBC, liver transplantation is still the most effective treatment. Accordingly, the alternative approaches, such as mesenchymal stem cell (MSC) transplantation, have been suggested as an effective alternative therapy for these patients. Due to their immunomodulatory characteristics, MSCs are considered as promising therapeutic agents for the therapy of autoimmune liver diseases, including PBC. In this review, we have summarized the therapeutic potential of MSCs for the treatment of these diseases, emphasizing molecular and cellular mechanisms responsible for MSC-based effects in an animal model of PBC and therapeutic potential observed in recently conducted clinical trials. We have also presented several outstanding problems including safety issues regarding unwanted differentiation of transplanted MSCs which limit their therapeutic use. Efficient and safe MSC-based therapy for PBC remains a challenging issue that requires continuous cooperation between clinicians, researchers, and patients.

## 1. Introduction

Primary biliary cholangitis (PBC) is an idiopathic chronic autoimmune cholestatic liver disease characterized by the progressive granulomatous destruction of small- and medium-sized intralobular and septal intrahepatic bile ducts with resultant cholestasis and progressive fibrosis [1, 2]. Although fatigue and pruritus are the most common symptoms of PBC, the disease begins quietly and for many years is manifested only by weakness, malaise, daytime somnolence, and low working efficiency [1]. Accordingly, it is important to elucidate the molecular and cellular mechanisms involved in the etiology and pathogenesis of PBC in order to prevent the development of inflammation and irreversible cirrhosis.

Despite the fact that a huge number of preclinical and clinical studies extensively investigated the natural history of PBC [3–10], etiology of PBC is still unknown. In recent years, it has become univocally accepted that an inappropriately activated immune response plays a crucial role in development and progression of PBC [1, 2, 6]. Current disease models envisage a T cell-driven biliary injury, resulting in secondary cholestasis, which arises on the background of combined genetic and environmental risks including infection [6]. It is believed that, in patients who had genetic predisposition to PBC, viruses [7, 8], bacteria [1], and xenobiotics [9, 10] either directly induce apoptosis of biliary epithelial cells (BECs) or trigger immune response against BECs as a result of molecular mimicry. Epitope of the E2 subunit of the pyruvate dehydrogenase complex (PDC: PDC-E2) autoantigen can be derived from microbes that utilize the PDC enzyme or, alternatively, from native proteins that were modified and become immunogenic by environmental xenobiotics/chemical compounds [2, 3]. In PBC, mitochondrial PDC-E2 autoantigen is the main target of immune response mediated by PDC-E2-specific helper CD4+ and cytotoxic CD8+ T cells accompanied with circulating PDC-E2 autoantibodies [3]. Although the serological hallmark of PBC remains the presence of antibodies to PDC-E2, autoreactive CD4+ T cells and CD8+ T cells have a central role in the pathogenesis of PBC [2, 11]. During the earliest stage of the diseases, IFN-*γ*-producing T cells (Th1 cells) are found in significantly higher number in the livers, periductular spaces, and peripheral blood of PBC patients while during the late stage of PBC majority of autoreactive CD4+ T cells produce IL-17 and IL-23 (Th17 cells) [2, 3, 12–15]. Importantly, when compared to healthy controls, patients with PBC display a relative reduction of circulating CD4+CD25+FoxP3+ T regulatory cells (Tregs) that play a critical role in immunosuppression, self-tolerance, and the prevention of autoimmune disease [16, 17]. The cytokine signature associated with PBC is also indicative of CD4+ T cell activation with a Th1/Th17 bias [18–21]. Serum levels of interleukin (IL)-18—which acts to release IL-12 and activate the Th1 pathway—and consequent release of IFN-*γ* from CD4+ T cells are elevated in PBC patients compared to healthy controls [18, 19]. Immunohistochemical studies support these observations, with PBC liver samples showing strong staining for IFN-*γ* with a shift to increased IL-23 and IL-17 staining in the later stage of the disease, accompanied with increased Th17 : Treg ratio in peripheral blood [20, 21].

Until 2016, ursodeoxycholic acid (UDCA) was, for more than two decades, the only US Food and Drug Administration- (FDA-) approved drug for the treatment of PBC [22]. UDCA increases the bile acid saturation in bile, resulting in increased bile acid clearance from the blood and reduced cholestatic symptoms, specifically pruritus. Additionally, UDCA has anti-inflammatory and immunomodulatory properties and protects hepatocytes from bile acid-induced apoptosis [22]. Nevertheless, more than 40% of PBC patients incompletely respond to UDCA treatment or are intolerant to UDCA, resulting with disease progression [23]. Most recently, results obtained in clinical studies [24, 25] demonstrated beneficent therapeutic effects of obeticholic acid (OCA) in the therapy of PBC and were the basis for the US FDA's approval of OCA for the treatment of PBC patients with incomplete response to UDCA [22]. OCA increases bile flow in cholestatic conditions and, through the activation of farnesoid X receptor (FXR), inhibits the uptake of bile acids, thereby protecting the hepatocytes from accumulation of cytotoxic bile acids [22]. Additionally, OCA has anti-inflammatory and antifibrotic properties that contribute to its beneficent effects in the therapy of PBC.

Despite the promising results of UDCA- and OCA-based therapies, liver transplantation is still the most effective treatment modality for PBC patients with end-stage liver disease [22]. However, the use of liver transplantation is limited because of organ donor shortage, financial considerations, and the requirement for lifelong immunosuppression [22, 23]. Accordingly, an alternative approach, such as stem cell transplantation, has been suggested as an effective alternative therapy for the treatment of end-stage PBC patients. Among stem cells, mesenchymal stem cells (MSCs) are, due to their immunomodulatory characteristics, considered as promising therapeutic agents for the therapy of PBC.

## 2. MSCs: New Players in Cell-Based Therapy of Liver Diseases

MSCs are adult, fibroblast-like, multipotent cells that can be found in almost all postnatal organs and tissues, including the liver [26]. Previous studies have shown that human MSCs, derived from the bone marrow (BM-MSCs), adipose tissue (AT-MSCs), amniotic fluid (AF-MSCs), dental pulp (DP-MSCs), umbilical cord (UC-MSCs), and fetal lung (FL-MSCs), in the presence of growth factors, cytokines, and chemical compounds, could differentiate into hepatocytes [27–31]. Although MSC differentiation into hepatocytes has been demonstrated *in vitro*, it is still controversial whether MSC transplantation can completely regenerate liver *in vivo* [32]. The vast majority of recently published studies indicated that beneficent effects of MSCs in the treatment of acute and chronic liver diseases are mainly based on suppression of immune cells responsible for liver injury [32]. After intravenous administration, MSCs manage to engraft in injured livers [33]. Inflammatory cytokines (tumor necrosis factor alpha (TNF-*α*), interleukin (IL)-1, interferon gamma (IFN-*γ*)), released after liver damage and during inflammation, induce cell surface expression of adhesion molecules that mediate rolling and transmigration of MSCs into extracellular matrix [32–35]. Immediately after their engraftment in the liver, MSCs affect innate and adaptive immune responses in cell-to-cell contact-dependent (through the programmed death (PD) ligand: PD receptor interaction) and in a paracrine manner, via the secretion of a wide variety of different soluble factors [32]. As far as we know to date, the capacity of MSC to alter immune response is largely due to the production of soluble factors such as transforming growth factor-*β* (TGF-*β*), hepatic growth factor (HGF), nitric oxide (NO), indolamine 2,3-dioxygenase (IDO), IL-10, IL-6, leukocyte inhibitory factor (LIF), IL-1 receptor antagonist (IL-1Ra), galectins, tumor necrosis factor *α*-stimulated gene 6 (TSG-6), human leukocyte antigen-G (HLA-G), hemeoxygenase-1 (HO-1), and prostaglandin E2 (PGE2) [32].

**Figure 1:**
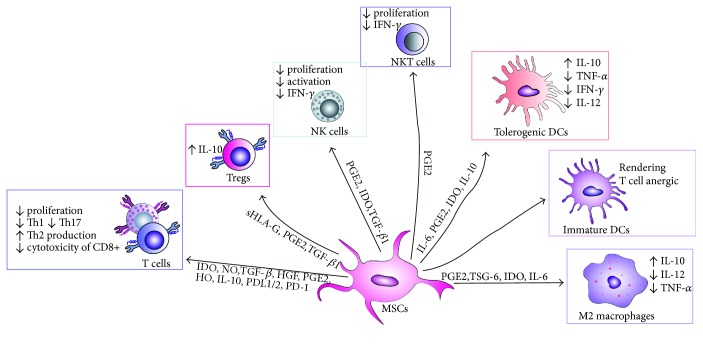
MSC-mediated suppression of immune cells. Through cell-to-cell contact or through producing of soluble factors, MSCs suppress proliferation of effector T cells, attenuate activation and cytokine production in NK and NKT cells, suppress maturation and activation of DCs, and promote the development of anti-inflammatory M2 macrophages. MSCs: mesenchymal stem cells; IFN-*γ*: interferon gamma; TNF*α*: tumor necrosis factor alpha; DCs: dendritic cell; NK; natural killer; PD-1: programmed cell death protein-1; PD-L1/2: programmed death-ligand 1/2; IDO: indoleamine 2,3-dioxygenase; NO: nitric oxide, TGF-*β*: transforming growth factor-*β*; HGF: hepatocyte growth factor; PGE2: prostaglandin E2; HO: hemeoxygenase; IL-10: interleukin 10; IL-6: interleukin 6; IL-12: interleukin 12; TSG-6: TNF-*α*-stimulated gene/protein 6; sHLA-G: soluble human leukocyte antigen-G.

MSCs suppress both innate and adaptive immunity in the liver [32, 33, 35]. Among innate immune cells, MSCs modulate function and cytokine profile of macrophages, dendritic cells, and natural killer (NK) and natural killer T (NKT) cells (Figure 1).

Classically activated (M1) macrophages (stimulated by Toll-like receptor (TLR) ligands and IFN-*γ*) produce high levels of proinflammatory cytokines (IL-12, IL-1, and TNF-*α*), reactive nitrogen, and oxygen species and are implicated in initiating and sustaining inflammation in acute liver inflammation [36]. In contrast, alternatively activated TGF-*β* and IL-10-producing (M2) macrophages have anti-inflammatory and reparative functions in acute liver damage [37]. As a result, a switch from the M1 to M2 phenotype is crucial for resolution of inflammation and tissue remodeling. MSC-mediated polarization of resident macrophages from classic M1 proinflammatory phenotype toward the anti-inflammatory M2 phenotype is dependent on both cellular contact and secretion of soluble factors, including PGE_2_, TSG-6, IL-6, and IDO [32, 33].

Immature DCs constantly enter the liver from the blood and preferentially induce tolerance in the liver [38]. Accordingly, the activation and consequent maturation of DCs seem to be the critical initial trigger for the onset of T- and NKT cell-mediated acute liver inflammation [39]. Immediately after hepatocyte damage and/or entrance of microbes, DCs infiltrate the liver, capture antigens, increase expression of costimulatory (CD40, CD80, and CD86), major histocompatibility complex (MHC), and CD1d molecules on their surface and activate T and NKT cells [38, 39]. DCs need an additional early signal from the innate immune system, particularly from liver NKT cells, to mature and gain competence to prime immune response in the liver [40]. MSCs, particularly through the production of prostaglandin E2 (PGE2), NO, and IDO, inhibit activation of liver NKT cells and consequent maturation of DCs, attenuate their capacity for antigen presentation, and alter their secretion profile resulting in increased production of anti-inflammatory IL-10 and decreased production of proinflammatory TNF-*α*, IFN-*γ*, and IL-12 [32, 33, 35, 41–47]. Immature DCs generated in the presence of MSCs do not express MHCII, CD40, CD80, and CD86 molecules and are able to render T helper-type 1 (Th1) cell anergic [32].

MSCs can directly suppress proliferation and expansion of T cells in PGE2, NO, and IDO dependent manner. The suppressive effects of PGE2 on the activation and expansion of T cells include the direct inhibitory effects on IL-2 production and the expression of IL-2 receptor and janus kinase (JAK)-3 which mediate the responsiveness of T cells to IL-2 [48]. Interestingly, production of PGE2 by MSCs and consequent suppression of T cell proliferation is significantly enhanced after MSC stimulation by TNF-*α* or IFN-*γ*, while inhibition of these cytokines resulted in the restoration of T cell proliferation [48]. TNF-*α* or IFN-*γ* provoke MSCs to express iNOS [49] and to produce NO which, in turn, increase IDO activity [50] and augment MSC-based suppression of immune response [33]. Thus, in an inflammatory microenvironment, in the presence of TNF-*α* or IFN-*γ*, MSCs produce large amounts of IDO that degrades tryptophan to kynurenine and other toxic metabolites (quinolinic acid and 3-hydroxy-anthranilic acid) [51]. Tryptophan starvation directly inhibits T cell proliferation, while IDO catabolites such as kynurenine and oxygen radicals induce apoptosis of T cells [51]. Furthermore, MSC-derived IDO inhibits the generation of cytotoxic CD8+ T cells (CTLs) and attenuate their cytotoxicity [52].

In addition to the suppression of T cell proliferation, MSCs may alter the cytokine profile of CD4+ T cells by decreasing the production of Th1 and Th17 cytokines (IFN-*γ* and IL-17) and by increasing the production of Th2 cytokines IL-4 and IL-10 [32]. Moreover, through the production of NO, IDO, IL-10, and TGF-*β*, MSCs can inhibit proliferation of Th1 and Th17 cells and may increase the number of CD4+CD25+FoxP3+ Tregs which suppress immune response and inflammation [53].

Since T cells play a central role in the pathogenesis of PBC, molecular mechanisms involved in MSC-based suppression of inflammatory T cells and expansion of Tregs were used as a starting point for a design of preclinical studies that investigated the therapeutic potential of MSCs in the treatment of PBC.

## 3. Therapeutic Effects of MSCs in PBC: What Have We Learnt from the Animal Model?

Several years ago, Wang and coworkers were first to demonstrate the beneficent effects of the bone marrow-derived mouse MSCs (BM-MSCs) in the treatment of polyinosinic-polycytidylic acid sodium- (PolyI:C-) induced mouse model of PBC [54]. PolyI:C is an IFN-*α* inducer that, after multiple intraperitoneal injections (5 mg/kg body weight, twice per week for 16 weeks), elevates Fas-associated death domain-like interleukin-1-b-converting enzyme inhibitory protein L (FLIPL, an anti-apoptotic protein) in hepatic CD4+ T cells of C57Bl/6 mice, which contributes to the aggravation of portal area inflammation, as seen in human PBC [55, 56].

In a study conducted by Wang et al. [54], BM-MSCs were intravenously injected (1 × 10^6^ cells) in C57Bl/6 mice, 16 weeks after the first injection of PolyI:C, and therapeutic effects of MSCs were evaluated 6 weeks later. BM-MSC treatment significantly attenuated serum levels of alkaline phosphatase (ALP) and antimitochondrial antibodies (AMA) and markedly decreased infiltration of mononuclear cells in the livers. Massive infiltration of lymphocytes and plasma cells in the bile duct epithelium cells, accompanied with the destruction of the basement membrane around bile ducts, was noticed in PolyI:C-treated mice. On the contrary, only a few mononuclear cells infiltrated the bile duct epithelium of PolyI:C + MSC-treated mice indicating that BM-MSCs suppressed the expansion and liver infiltration of immune cells [54].

**Figure 2:**
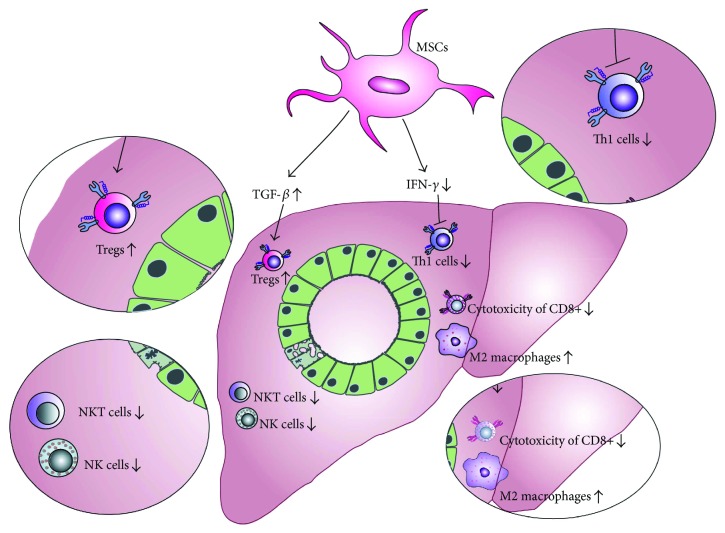
MSC-based attenuation of immune response in PBC. MSCs modulate immune response and attenuate PBC by producing TGF-*β* that resulted in an increased expansion of Tregs and anti-inflammatory M2 macrophages. Additionally, MSCs attenuate production of inflammatory cytokines, particularly IFN-*γ*, and suppress Th1 immune response including inhibition of IFN-*γ*-producing CD4+Th1 cells, cytotoxic CD8+ T lymphocytes, NK, and NKT cells.

Additionally, PolyI:C-treated mice that received BM-MSCs had attenuated serum levels of inflammatory cytokines, particularly IFN-*γ* when compared to BM-MSC-untreated animals, indicating that BM-MSCs suppressed Th1 immune response in the injured livers (Figure 2) [54, 55]. Importantly, BM-MSC therapy significantly increased concentration of immunosuppressive TGF-*β* in sera of PolyI:C-treated mice. Elevated levels of TGF-*β* correlated with an increased number of immunosuppressive Tregs in peripheral blood and mesenteric lymph nodes indicating that BM-MSC attenuation of PolyI:C-induced PBC was a consequence of MSC-dependent expansion of Tregs (Figure 2).

Tregs are crucial for the establishment and maintenance of immunologic self-tolerance in the liver and are a major population of immunosuppressive immune cells that prevent progression of autoimmune liver diseases, including PBC [16, 17]. It is well known that MSCs produce large amounts of TGF-*β* upon liver injury [32] and that MSC-derived TGF-*β* is important for differentiation and expansion of immunosuppressive Tregs [53]. A knockdown of TGF-*β* in MSCs before coculturing them with peripheral blood mononuclear cells showed no significant increase in Treg frequency in the cocultures [57]. Since TGF-*β* was thought to be a vital antifibrotic MSC-derived factor in the disordered liver environment [58], Wang et al. concluded that BM-MSC-dependent augmentation of TGF-*β* pathway may be involved in the beneficent effects of BM-MSC in the therapy of PolyI:C-induced PBC and proposed MSC-derived TGF-*β* and Treg interplay as a potentially new therapeutic approach for the clinical use of MSCs in the therapy of patients with PBC.

## 4. Therapeutic Effects of MSCs in the Treatment of Patients with PBC

Until now, only two clinical studies, both conducted in China, investigated the therapeutic potential of MSCs in the treatment of patients suffering from PBC [59, 60].

The first of these two clinical trials was conducted in Research Center for Biological Therapy, Beijing 302 Hospital and was registered at https://ClinicalTrials.gov of the National Institutes of Health of the USA (registration number NCT01662973). Seven PBC patients with an incomplete response to UDCA (that did not have a normalization of their ALP after a minimum of six months of treatment with adequate doses of UDCA) were enrolled in the study with the aim to evaluate safety and efficacy of umbilical cord-derived MSCs (UC-MSCs) in the therapy of PBC [59].

UC-MSCs have been chosen because of their potential to induce expansion of Tregs and to suppress clonal expansion of activated T cells [26, 61]. Derivation of MSCs from UC is a noninvasive and safe procedure. Under standard culture conditions, UC-MSC showed quick expansion and short generation time enabling large yields of these cells. Low expression of MHC molecules and potent immunomodulatory characteristics make UC-MSCs useful for allogeneic transplantations [62]. Differences between UC-MSCs and MSCs from other tissues could be observed concerning the success rate of isolating, proliferation capacity, and clonality. In contrast to BM-MSCs, UC-MSCs have the highest rates of cell proliferation and clonality and significantly lower expression of p53, p21, and p16, well-known markers of senescence [63, 64].

In this study, UC-MSCs (5 × 10^5^ cells/kg body weights) were infused through a peripheral vein and were given three times at 4-week intervals in combination with standard UDCA therapy [59]. All the seven patients tolerated the UC-MSC treatment well. After 48 weeks of follow-up, no obvious short-term side-effect (such as skin rash, infection, coma, and shock) or long-term complications (such as upper gastrointestinal hemorrhage, and hepatic encephalopathy) were noticed in patients that received UC-MSCs indicating that multiple injections of UC-MSCs was a safe therapeutic approach.

The combined treatment of UC-MSC and UDCA led to a significant decrease in serum ALP and *γ*-glutamyltransferase (GGT) levels at the end of the follow-up period as compared with baseline, while other biochemical parameters in sera (total bilirubin, albumin, aspartate aminotransferase (AST), prothrombin time activity, and international normalized ratio) were not significantly reduced. UC-MSC treatment did not affect serum concentration of immunoglobulin (Ig) M, IgA, IgG, or AMA and longer follow-up studies might be required to confirm the beneficial effect of UC-MSC treatment on autoantibody-producing plasma cells.

UC-MSC therapy can improve the quality of life of PBC patients. Symptoms, most usually seen in PBC patients, such as fatigue and pruritus, were alleviated in most patients that received UC-MSC treatment [59]. Although this data is encouraging, it should be noted that these observations still need confirmation. Future studies should quantify fatigue and pruritus using more objective measures, such as the fatigue impact score and the PBC-40 fatigue domain score for fatigue and the 5-D itch scale and the visual analog scale for pruritus. It is important to highlight limitations of this study: a small number of enrolled patients and the fact that the mechanism of UC-MSC-based effects were not explored.

Next, the clinical study conducted by Wang and colleagues in Peking Union Medical College Hospital tried to fulfill these expectations [60]. The objectives of this study were to evaluate the safety and efficacy of allogenic BM-MSC (obtained from healthy family donors of PBC patients) and to determine the mechanisms involved in their therapeutic effects in UDCA-resistant patients.

BM-MSCs were chosen for this study because of properties that enable their therapeutic use such as easy acquisition, quick proliferation in vitro, low surface expression of major histocompatibility complex (MHC) antigens and minor immunological rejection, long-term coexistence in the host, maintenance of differentiation potential after repeated passages, and ease of transplantation [64, 65]. However, the derivation of BM-MSCs involves harvesting of BM which is a highly invasive procedure and the number and maximal lifespan of BM-MSCs significantly decline by aging [66] that may limit their clinical application.

Ten patients were enrolled in this trial. They intravenously received 3–5 × 10^5^ BM-MSCs/kg body weight. All patients were permitted to concurrently continue their previous UDCA treatment. The safety assessments included vital signs and discomfort recordings during BM-MSC infusion and 1, 6, 12, 24, 48, and 72 h after. Vital signs of the patients remained stable during a 72 h observation, and no adverse events were observed indicating that intravenous injection of BM-MSCs is a safe procedure.

The efficacy of BM-MSCs in UDCA-resistant PBC was evaluated at 1, 3, 6, and 12 months after transplantation of BM-MSCs. The life quality of the patients was improved after transplantation of BM-MSCs as demonstrated by the responses to the PBC-40 questionnaire. Biochemical parameters and histological analysis confirmed these findings. Serum levels of alanine aminotransferase (ALT), AST, GGT, and IgM significantly decreased from baseline after injection of BM-MSCs. Histological liver deterioration such as fibrosis was not noticed in PBC patients that received BM-MSCs [60].

Similarly, as it was observed in the animal model of PBC [54], this study also documented the expansion of Tregs in peripheral blood of BM-MSC-treated patients with PBC, further confirming the importance of Tregs for beneficial effects of BM-MSCs in the therapy of PBC. Increased proliferation of Tregs was accompanied with an increased presence of Tregs in the injured livers and with a reduced number of inflammatory cytotoxic CD8+ T cells in peripheral blood and in the liver. These alterations in CD8+ T cells and Tregs were followed by elevated serum levels of IL-10, one of the most important MSC-derived immunoregulatory and anti-inflammatory cytokines [32] indicating that modulation of immune response has a crucial role in MSC-mediated attenuation of PBC.

Importantly, the optimal therapeutic effect of transplanted BM-MSCs was observed at 3 to 6 months after their injection and could be maintained for 12 months. Nevertheless, further studies are required to determine the optimal frequency of BM-MSC infusions and to evaluate the safety of MSC-based therapy in long-term follow-up.

## 5. Challenges towards Clinical Use of MSCs in the Therapy of PBC

Although MSCs have been already used in two clinical studies and obtained results were promising, there are still several challenges that should be addressed with the aim to improve their therapeutic potential in the therapy of PBC.

First, immunosuppressive characteristics of MSCs should be thoroughly analyzed and considered in future clinical trials in order to avoid potential undesirable interactions with the immunomodulatory drugs that are used as standard therapy in PBC treatment.

Second, an optimal number of transplanted MSCs and optimal frequency of MSC injections should be clearly defined with the aim to find the right balance between safety and effectiveness of MSC-based therapy of PBC.

Finally, safety issues regarding MS-based therapy of PBC is still a matter of debate, especially in the long-term follow-up. The primary concern is unwanted differentiation of the transplanted MSCs into undesired tissues, including bone and cartilage. Local microenvironment in which MSCs engraft contains factors that induce unwanted differentiation of transplanted MSCs *in vivo*. For example, encapsulated structures that contained calcifications or ossifications were found in the infarcted areas of the myocardium after transplantation of MSCs [67, 68]. Therefore, new research and preclinical studies should be focused in the definition of factors and signaling pathways that are responsible for the fate of MSCs after their *in vivo* administration.

## 6. Conclusions

Because of their immunomodulatory properties, MSCs are considered as new therapeutic agents for the treatment of PBC. Results obtained in many preclinical and in two clinical studies suggest that intravenous application of BM-MSCs or UC-MSCs is safe and beneficial therapeutic approaches for the treatment of UDCA-resistant patients with PBC.

Immediately after intravenous injection, MSCs engraft in the livers and create anti-inflammatory microenvironment by attenuating production of inflammatory cytokines in liver-infiltrated T cells, macrophages, and NK cells. Additionally, through the production of TGF-*β*, MSCs promote the expansion of immunosuppressive Tregs and M2 macrophages which in turn, in IL-10-dependent manner, inhibit activation of helper CD4+ T cells and suppress cytotoxicity of CD8+ T lymphocytes, NK, and NKT cells, resulting with the attenuation of PBC.

Nevertheless, it should be noted that both clinical trials that demonstrated beneficent effects of MSCs in PBC treatment recruited a small number of patients. Accordingly, the optimal origin, number, and frequency of transplanted MSCs as well as safety of MSC-based therapy still have to be confirmed in long-term follow-up clinical trials with higher numbers of enrolled patients. To address this concern, future clinical studies have to be conducted with the aim to utterly exploit the promising therapeutic potential of MSCs in the treatment of PBC.

## References

[1] Reshetnyak V. I. (2015). Primary biliary cirrhosis: clinical and laboratory criteria for its diagnosis. *World Journal of Gastroenterology*.

[2] Webb G. J., Siminovitch K. A., Hirschfield G. M. (2015). The immunogenetics of primary biliary cirrhosis: a comprehensive review. *Journal of Autoimmunity*.

[3] Hirschfield G. M., Gershwin M. E. (2013). The immunobiology and pathophysiology of primary biliary cirrhosis. *Annual Review of Pathology: Mechanisms of Disease*.

[4] Joshita S., Umemura T., Tanaka E., Ota M. (2017). Genetic contribution to the pathogenesis of primary biliary cholangitis. *Journal of Immunology Research*.

[5] Hsueh Y. H., Chang Y. N., Loh C. E., Gershwin M. E., Chuang Y. H. (2016). AAV-IL-22 modifies liver chemokine activity and ameliorates portal inflammation in murine autoimmune cholangitis. *Journal of Autoimmunity*.

[6] Kouroumalis E., Notas G. (2015). Primary biliary cirrhosis: from bench to bedside. *World Journal of Gastrointestinal Pharmacology and Therapeutics*.

[7] Maroni L., Hohenester S. D., van de Graaf S. F. J. (2017). Knockout of the primary sclerosing cholangitis-risk gene *Fut2* causes liver disease in mice. *Hepatology*.

[8] Floreani A., Mangini C., Reig A. (2017). Thyroid dysfunction in primary biliary cholangitis: a comparative study at two European centers. *The American Journal of Gastroenterology*.

[9] Arsenijevic A., Milovanovic M., Milovanovic J. (2016). Deletion of galectin-3 enhances xenobiotic induced murine primary biliary cholangitis by facilitating apoptosis of BECs and release of autoantigens. *Scientific Reports*.

[10] Lleo A., Bian Z., Zhang H. (2016). Quantitation of the Rank-Rankl axis in primary biliary cholangitis. *PLoS One*.

[11] Nakagawa R., Muroyama R., Saeki C. (2017). miR-425 regulates inflammatory cytokine production in CD4^+^ T cells via N-Ras upregulation in primary biliary cholangitis. *Journal of Hepatology*.

[12] Tsuneyama K., Baba H., Morimoto Y., Tsunematsu T., Ogawa H. (2017). Primary biliary cholangitis: its pathological characteristics and immunopathological mechanisms. *The Journal of Medical Investigation*.

[13] Zhou Z. Q., Tong D. N., Guan J. (2017). Circulating follicular helper T cells presented distinctively different responses toward bacterial antigens in primary biliary cholangitis. *International Immunopharmacology*.

[14] Liang D. Y., Hou Y. Q., Luo L. J., Ao L. (2016). Altered expression of miR-92a correlates with Th17 cell frequency in patients with primary biliary cirrhosis. *International Journal of Molecular Medicine*.

[15] Shi T. Y., Zhang T., Zhang L. N., Yang Y. J., Zhang H. Z., Zhang F. C. (2015). The distribution and the fibrotic role of elevated inflammatory Th17 cells in patients with primary biliary cirrhosis. *Medicine*.

[16] Wang Y. H., Yang W., Yang J. B. (2015). Systems biologic analysis of T regulatory cells genetic pathways in murine primary biliary cirrhosis. *Journal of Autoimmunity*.

[17] Wang L., Wang F. S., Chang C., Gershwin M. (2014). Breach of tolerance: primary biliary cirrhosis. *Seminars in Liver Disease*.

[18] Limongi F. (2015). Th1 cytokines and chemokines in primary biliary cirrhosis. *La Clinica Terapeutica*.

[19] Yamano T. (2000). Serum interferon-gamma-inducing factor/IL-18 levels in primary biliary cirrhosis. *Clinical & Experimental Immunology*.

[20] Yang C. Y. (2014). IL-12/Th1 and IL-23/Th17 biliary microenvironment in primary biliary cirrhosis: implications for therapy. *Hepatology*.

[21] Rong G. (2009). Imbalance between T helper type 17 and T regulatory cells in patients with primary biliary cirrhosis: the serum cytokine profile and peripheral cell population. *Clinical & Experimental Immunology*.

[22] Jhaveri M., Kowdley K. (2017). New developments in the treatment of primary biliary cholangitis - role of obeticholic acid. *Therapeutics and Clinical Risk Management*.

[23] Melchor-Mendoza Y. K., Martínez-Benítez B., Mina-Hawat A., Rodríguez-Leal G., Duque X., Moran-Villota S. (2017). Ursodeoxycholic acid therapy in patients with primary biliary cholangitis with limited liver transplantation availability. *Annals of Hepatology*.

[24] Hirschfield G. M., Mason A., Luketic V. (2015). Efficacy of obeticholic acid in patients with primary biliary cirrhosis and inadequate response to ursodeoxycholic acid. *Gastroenterology*.

[25] Nevens F., Andreone P., Mazzella G. (2016). A placebo-controlled trial of obeticholic acid in primary biliary cholangitis. *The New England Journal of Medicine*.

[26] Volarevic V., Ljujic B., Stojkovic P., Lukic A., Arsenijevic N., Stojkovic M. (2011). Human stem cell research and regenerative medicine—present and future. *British Medical Bulletin*.

[27] Lee C. W., Huang W. C., Huang H. D. (2017). DNA methyltransferases modulate hepatogenic lineage plasticity of mesenchymal stromal cells. *Stem Cell Reports*.

[28] Fu Y., Deng J., Jiang Q. (2016). Rapid generation of functional hepatocyte-like cells from human adipose-derived stem cells. *Stem Cell Research & Therapy*.

[29] Zheng Y., Gao Z., Xie C. (2008). Characterization and hepatogenic differentiation of mesenchymal stem cells from human amniotic fluid and human bone marrow: a comparative study. *Cell Biology International*.

[30] Ishkitiev N., Yaegaki K., Calenic B. (2010). Deciduous and permanent dental pulp mesenchymal cells acquire hepatic morphologic and functional features *in vitro*. *Journal of Endodontia*.

[31] Ling L., Ni Y., Wang Q. (2008). Transdifferentiation of mesenchymal stem cells derived from human fetal lung to hepatocyte-like cells. *Cell Biology International*.

[32] Volarevic V., Nurkovic J., Arsenijevic N., Stojkovic M. (2014). Concise review: therapeutic potential of mesenchymal stem cells for the treatment of acute liver failure and cirrhosis. *Stem Cells*.

[33] Gazdic M., Simovic Markovic B., Vucicevic L. (2017). Mesenchymal stem cells protect from acute liver injury by attenuating hepatotoxicity of liver natural killer T cells in an inducible nitric oxide synthase- and indoleamine 2,3-dioxygenase-dependent manner. *Journal of Tissue Engineering and Regenerative Medicine*.

[34] Nitzsche F., Müller C., Lukomska B., Jolkkonen J., Deten A., Boltze J. (2017). Concise review: MSC adhesion cascade—insights into homing and transendothelial migration. *Stem Cells*.

[35] Milosavljevic N., Gazdic M., Markovic B. S. (2017). Mesenchymal stem cells attenuate acute liver injury by altering ratio between interleukin 17 producing and regulatory natural killer T cells. *Liver Transplantation*.

[36] Kakinuma Y., Kimura T., Watanabe Y. (2017). Possible involvement of liver resident macrophages (Kupffer cells) in the pathogenesis of both intrahepatic and extrahepatic inflammation. *Canadian Journal of Gastroenterology and Hepatology*.

[37] Volarevic V., Milovanovic M., Ljujic B. (2012). Galectin-3 deficiency prevents concanavalin A–induced hepatitis in mice. *Hepatology*.

[38] Doherty D. G. (2016). Immunity, tolerance and autoimmunity in the liver: a comprehensive review. *Journal of Autoimmunity*.

[39] Volarevic V., Markovic B. S., Bojic S. (2015). Gal-3 regulates the capacity of dendritic cells to promote NKT-cell-induced liver injury. *European Journal of Immunology*.

[40] Wang J., Cao X., Zhao J. (2017). Critical roles of conventional dendritic cells in promoting T cell-dependent hepatitis through regulating natural killer T cells. *Clinical & Experimental Immunology*.

[41] Spaggiari G. M., Moretta L. (2013). Cellular and molecular interactions of mesenchymal stem cells in innate immunity. *Immunology and Cell Biology*.

[42] Wang Q., Ding G., Xu X. (2016). Immunomodulatory functions of mesenchymal stem cells and possible mechanisms. *Histology and Histopathology*.

[43] Dokic J. M., Tomic S. Z., Colic M. J. (2016). Cross-talk between mesenchymal stem/stromal cells and dendritic cells. *Current Stem Cell Research & Therapy*.

[44] Higashimoto M., Sakai Y., Takamura M. (2013). Adipose tissue derived stromal stem cell therapy in murine ConA-derived hepatitis is dependent on myeloid-lineage and CD4^+^ T-cell suppression. *European Journal of Immunology*.

[45] Najar M., Raicevic G., Fayyad-Kazan H., Bron D., Toungouz M., Lagneaux L. (2016). Mesenchymal stromal cells and immunomodulation: a gathering of regulatory immune cells. *Cytotherapy*.

[46] Kariminekoo S., Movassaghpour A., Rahimzadeh A., Talebi M., Shamsasenjan K., Akbarzadeh A. (2016). Implications of mesenchymal stem cells in regenerative medicine. *Artificial Cells, Nanomedicine, and Biotechnology*.

[47] Zachar L., Bačenková D., Rosocha J. (2016). Activation, homing, and role of the mesenchymal stem cells in the inflammatory environment. *Journal of Inflammation Research*.

[48] Kalinski P. (2012). Regulation of immune responses by prostaglandin E_2_. *Journal of Immunology*.

[49] Li W., Ren G., Huang Y. (2012). Mesenchymal stem cells: a double-edged sword in regulating immune responses. *Cell Death & Differentiation*.

[50] Chae H. K., Song W. J., Ahn J. O. (2017). Immunomodulatory effects of soluble factors secreted by feline adipose tissue-derived mesenchymal stem cells. *Veterinary Immunology and Immunopathology*.

[51] Orabona C., Grohmann U. (2010). Indoleamine 2,3-dioxygenase and regulatory function: tryptophan starvation and beyond. *Methods in Molecular Biology*.

[52] Li M., Sun X., Kuang X., Liao Y., Li H., Luo D. (2014). Mesenchymal stem cells suppress CD8^+^ T cell-mediated activation by suppressing natural killer group 2, member D protein receptor expression and secretion of prostaglandin E_2_, indoleamine 2, 3-dioxygenase and transforming growth factor-β. *Clinical & Experimental Immunology*.

[53] English K., Ryan J. M., Tobin L., Murphy M. J., Barry F. P., Mahon B. P. (2009). Cell contact, prostaglandin E_2_ and transforming growth factor beta 1 play non-redundant roles in human mesenchymal stem cell induction of CD4^+^CD25^High^forkhead box P3^+^ regulatory T cells. *Clinical & Experimental Immunology*.

[54] Wang D., Zhang H., Liang J. (2011). Effect of allogeneic bone marrow–derived mesenchymal stem cells transplantation in a polyI:C-induced primary biliary cirrhosis mouse model. *Clinical and Experimental Medicine*.

[55] Okada C., Akbar S. M. F., Horiike N., Onji M. (2005). Early development of primary biliary cirrhosis in female C57BL/6 mice because of poly I:C administration. *Liver International*.

[56] Jiang T., Han Z., Chen S. (2009). Resistance to activation-induced cell death and elevated FLIP_L_ expression of CD4+ T cells in a polyI:C-induced primary biliary cirrhosis mouse model. *Clinical and Experimental Medicine*.

[57] Patel S. A., Meyer J. R., Greco S. J., Corcoran K. E., Bryan M., Rameshwar P. (2010). Mesenchymal stem cells protect breast cancer cells through regulatory T cells: role of mesenchymal stem cell-derived TGF-β. *Journal of Immunology*.

[58] Hong J. W., Lim J. H., Chung C. J. (2017). Immune tolerance of human dental pulp-derived mesenchymal stem cells mediated by CD4^+^CD25^+^FoxP3^+^ regulatory T-cells and induced by TGF-β1 and IL-10. *Yonsei Medical Journal*.

[59] Wang L., Li J., Liu H. (2013). A pilot study of umbilical cord-derived mesenchymal stem cell transfusion in patients with primary biliary cirrhosis. *Journal of Gastroenterology and Hepatology*.

[60] Wang L., Han Q., Chen H. (2014). Allogeneic bone marrow mesenchymal stem cell transplantation in patients with UDCA-resistant primary biliary cirrhosis. *Stem Cells and Development*.

[61] Floreani A., Sun Y., Zou Z. S. (2016). Proposed therapies in primary biliary cholangitis. *Expert Review of Gastroenterology & Hepatology*.

[62] Arutyunyan I., Elchaninov A., Makarov A., Fatkhudinov T. (2016). Umbilical cord as prospective source for mesenchymal stem cell-based therapy. *Stem Cells International*.

[63] Jin H., Bae Y., Kim M. (2013). Comparative analysis of human mesenchymal stem cells from bone marrow, adipose tissue, and umbilical cord blood as sources of cell therapy. *International Journal of Molecular Sciences*.

[64] Macrin D., Joseph J. P., Pillai A. A., Devi A. (2017). Eminent sources of adult mesenchymal stem cells and their therapeutic imminence. *Stem Cell Reviews*.

[65] Kim H. J., Park J. S. (2017). Usage of human mesenchymal stem cells in cell-based therapy: advantages and disadvantages. *Development & Reproduction*.

[66] Charif N., Li Y. Y., Targa L. (2017). Aging of bone marrow mesenchymal stromal/stem cells: implications on autologous regenerative medicine. *Bio-medical Materials and Engineering*.

[67] Breitbach M., Bostani T., Roell W. (2007). Potential risks of bone marrow cell transplantation into infarcted hearts. *Blood*.

[68] Yoon Y. S., Park J. S., Tkebuchava T., Luedeman C., Losordo D. W. (2004). Unexpected severe calcification after transplantation of bone marrow cells in acute myocardial infarction. *Circulation*.

